# Genome Announcement: The Draft Genomes of Two *Radopholus similis* populations from Costa Rica

**DOI:** 10.21307/jofnem-2019-052

**Published:** 2019-09-17

**Authors:** Catherine L. Wram, Cedar N. Hesse, Sulochana K. Wasala, Dana K. Howe, Amy B. Peetz, Dee R. Denver, Danny Humphreys-Pereira, Inga A. Zasada

**Affiliations:** 1 Department of Botany and Plant Pathology, Oregon State University, Corvallis, OR 97331; 2 USDA-ARS Horticultural Crops Research Unit, Corvallis, OR 97331; 3 Department of Integrative Biology, Oregon State University, Corvallis, OR 97331; 4 Laboratorio de Nematología, Centro de Investigación en Protección de Cultivos, Escuela de Agronomía, Universidad de Costa Rica, San Jose, Costa Rica, 2060

**Keywords:** burrowing nematode, draft genome, genomics, *Radopholus similis*

## Abstract

*Radopholus similis* is an economically important pest of both banana and citrus in tropical regions. Here we present draft genomes from two populations of *R. similis* from Costa Rica that were created and assembled using short read libraries from Illumina HiSeq technology.

The migratory endoparasite, *Radopholus similis*, is a pest to over 250 plant species, including economically important crops such as banana and citrus (Haegeman et al., 2010). Damage caused by *R. similis* feeding on roots can lead to secondary infections by fungi and bacteria, eventually leading to root collapse and in some instances, as with banana, plant toppling (Haegeman et al., 2010). With a wide geographical distribution including North and South America, the Caribbean, Africa, and Asia, there have been efforts to better understand the mechanisms behind this nematode’s ability to infect its hosts. These efforts include characterizing cell-wall degrading enzymes (Haegeman et al., 2008; Haegeman et al., 2009), sequencing of the mitochondrial genome (Jacob et al., 2009), and generating multi-stage transcriptomes (Jacob et al., 2008; Huang et al., 2019). The addition of genomes from multiple populations would be beneficial to the community characterizing this nematode.

*Radopholus similis* samples were collected from two different locations and hosts in Costa Rica. Nematodes were collected from plantain roots in La Virgen, Sarapiquí (Rv) and from bananas in Río Frío, Horquetas, Sarapiquí (Rd). After samples were collected, adult *R. similis* were randomly hand-picked from each sample. QIAmp DNA Micro Kit (Qiagen, Hilden, Germany) was used to extract DNA from 560 and 1,000 adults from the Rd and Rv populations, respectively. From these DNA extractions, genomic libraries were made using NEBNext Ultra II DNA Library Prep Kit for Illumina (San Diego, CA). Whole genome sequencing was performed on the Illumina HiSeq 3000 at the Center for Genome Research and Biocomputing at Oregon State University (Corvallis, OR). Raw reads were screened for sequencing adapters, trimmed and filtered for quality (*Q* = 20) using BBDuk (https://github.com/BioInfoTools/BBMap) and resulted in 5,376,812 and 14,006,275 paired-end 150 reads for Rd and Rv, respectively. Quality-filtered reads were assembled de novo using metaSPAdes (Nurk et al., 2017) and putative taxonomic origins of the resulting contigs were investigated using the Blob Tools workflow (Kumar et al., 2013). Briefly, individual contigs were assigned a phylum based on BLAST similarity (*E*-value < 10e−25) to sequences found in the NCBI “nt” database and visualized based on average read coverage and GC content (Kumar et al., 2013). To remove fungal and bacterial contamination, reads that were identified as belonging to contigs assigned to the phylum Nematoda or had no identity were then used to assemble the *R. similis* genome in the de novo assembler SPAdes Version 3.12 (Bankevich et al., 2012). Contigs in the assembly that were less than 500 bp were removed and QUAST was used to calculate genome assembly statistics (Gurevich et al., 2013).

The resulting genome assemblies for *R. similis* populations Rv and Rd were 50,532,728 and 50,089,881 bp in size, respectively (Table 1). The Rv assembly had approximately 1,000 contigs fewer than the Rd assembly, which had 6,195 contigs in total. The N50 for the two assemblies were similar in size; the Rv assembly had a N50 of 27,798 bp while the Rd assembly N50 was 20,071 bp. The largest contig in the Rd assembly was over 170,000 bp, while the largest contig in the Rv assembly was ~160,000 bp. Both assemblies had a GC% content of 47%, consistent with the GC content of the *R. similis* transcriptome, found to be 49% (Jacob et al., 2008).

**Table 1.** Comparison of the genome assemblies of *Radopholus similis* population collected from Costa Rica.

Assembly Statistic	Population (Rv)^a^	Population (Rd)^b^	ASM476467v1^c^
Size (bp)	**50,555,211**	**50,089,881**	65,195,707
Number of Scaffolds	5,194	6,195	8,133
Largest Scaffold (bp)	159,742	174,442	502,931
GC Content (%)	47.06	47.11	51.64
N50 Value (bp)	27,798	20,071	13,529
No. Contigs > 5,000 bp	2,081	2,476	3,451
No. Contigs > 10,000 bp	1,468	1,587	1,726
No. Contigs > 25,000 bp	618	499	419
No. Contigs > 50,000 bp	167	95	83
Complete BUSCOs	588 (59.9%)	593 (60.4%)	585 (59.5%)
Complete and single-copy BUSCOs	573 (58.4%)	577 (58.8%)	564 (57.4%)
Complete and duplicated BUSCOs	15 (1.5%)	16 (1.6%)	21 (2.1%)
Fragmented BUSCOs	93 (9.5%)	95 (9.7%)	85 (8.7%)
Missing BUSCOs	301 (30.6%)	294 (29.2%)	312 (31.8%)

The draft genomes from each *R. similis* population were assessed for completeness using BUSCO v. 3 (Simão et al., 2015). Of the 982 genes in the Nematoda dataset used to verify genome completeness, the *R. similis* Rd and Rv genomes had similar percentages of complete BUSCO genes with 60.5% and 59.4%, respectively. Most of these genes were single copies, with roughly 1.5% duplicated in both assemblies. Of these completed genes, 541 were shared between each assembly with 21 found only in the Rd assembly and 13 only in the Rv assembly. Each assembly also shared 74 fragmented BUSCO genes. In addition to verifying completeness using BUSCO genes, the existing mitochondrial genome for *R. similis* was obtained from GenBank (accession no. PRJNA40409) and was used as a BLAST query in each assembly. In both assemblies, a single contig was able to cover 100% of the mitochondrial genome query with *E*-values of 0 and percentage identities of 99% with soft masking parameters.

To improve each assemblies’ usefulness, unsupervised gene annotations were done for both the Rv and Rd assemblies. STAR (Dobin et al., 2013) was used to align transcriptomic data (Huang et al., 2019) from three *R. similis* life stages: adult female, juvenile, and egg (GenBank accessions: SRX3518251, SRX3518250, and SRX3518249) to each completed genome. Annotations were done using BRAKER1 (Hoff et al., 2016) with the mapped RNA-seq data and the completed genomes as input. The resulting predicted genes were filtered out if they did not include a start and stop codon. Both assemblies had a comparable number of predicted genes. The Rd assembly had 12,452 genes identified, while in the Rv assembly 13,120 genes were predicted.

A genome for *R. similis* is available on NCBI (assembly no. ASM476467v1). However, the genome statistics associated with the ASM476467v1 assembly suggests that there is a ~15 Mb difference in size with our *R. similis* Rd and Rv assemblies. In order to ensure that the Rd and Rv assemblies did not underestimate genome size, the MUMmer program nucmer (Kurtz et al., 2004) with default parameters was used to align the *R. similis* Rd assembly to the ASM476467v1 assembly to explore areas of each genome that did not align. There were 3,671 contigs from the ASM476467v1 assembly that did not align to the Rd assembly, representing 18,677,081 bp. In the Rd assembly, there were 495 contigs that did not align to the ASM476467v1 assembly, or 4,473,793 bp. Contigs that were unaligned from both assemblies were identified using BLAST as non-nematode, indicating there was likely human, bacterial, and fungal contamination, explaining the difference in assembly size. The GC content of the 3,671 contigs of the ASM476467v1 that did not align with the Rd assembly was 57%, while the GC content without those contigs was 49%. The high GC content in the non-matching portion of the ASM476467v1 assembly, or the portion found to be potential contamination, could therefore be skewing the overall GC percentage of the assembly. The contigs in the Rd assembly that did not match ASM476467v1 assembly had a GC content of 55%, while the Rd assembly without these 495 contigs was 47%. Therefore, it is unlikely that this latter set of contigs have much influence in overall GC content.

Using D-GENIES (Cabanettes and Klopp, 2018), both the Rd and Rv assemblies were compared for synteny to the ASM476467v1 assembly. Aligned to the Rd assembly, 66.43% of the ASM476467v1 assembly matched with greater than 75% identity and 33.55% of the ASM476467v1 assembly was not matched. When the ASM476467v1 assembly was compared to Rv, 58.76% of the assembly aligned with greater than 75% identity, while 41.21% went unmatched. There was a total of 43 Mb from the ASM476467v1 assembly that matched with greater than 75% identity to the Rd assembly and 38 Mb that matched the Rv assembly.

The Rd and Rv assemblies are uploaded to NCBI (NCBI BioProject PRJNA541590, accession no. VAHI00000000 and VAHH00000000). The addition of these two genome sequences from different Costa Rican populations of *R. similis* will be an additional resource for the nematology community and could provide tools for future work in nematode comparative genomics, population-level studies, and exploration of effector genes occurring in this nematode.

## References

[ref001] Bankevich A., Nurk S., Antipov D., Gurevich A. A., Dvorkin M., Kulikov A. S., V. M Lesin, Nikolenko S. I., Pham S., Prjibelski A. D., Pyshkin A. V., Sirotkin A. V., Vyahhi N., Tesler G., Alekseyev M. A. and Pevzner P. A. 2012 SPAdes: A new genome assembly algorithm and its applications to single-cell sequencing. Journal of Computational Biology 19:455–77. 22506599 10.1089/cmb.2012.0021 PMC3342519

[ref002] Cabanettes F. and Klopp C. 2018 D-GENIES: dot plot large genomes in an interactive, efficient and simple way. PeerJ 6:e4958. 29888139 10.7717/peerj.4958 PMC5991294

[ref003] Dobin A., Davis C. A., Schlesinger F., Drenkow J., Zaleski C., Jha S., Batut P., Chaisson M. and Gingeras T. R. 2013 STAR: ultrafast universal RNA-seq aligner. Bioinformatics 29:15–21. 23104886 10.1093/bioinformatics/bts635 PMC3530905

[ref004] Gurevich A., Saveliev V., Vyahhi N. and Tesler G. 2013 QUAST: quality assessment tool for genome assemblies. Bioinformatics 29:1072–5. 23422339 10.1093/bioinformatics/btt086 PMC3624806

[ref005] Haegeman A., Elsen A., Waele D. D. and Gheysen G. 2010 Emerging molecular knowledge on *Radopholus similis*, an important nematode pest of banana. Molecular Plant Pathology 11:315–23. 20447280 10.1111/j.1364-3703.2010.00614.x PMC6640332

[ref006] Haegeman A., Jacob J., Vanholme B., Kyndt T. and Gheysen G. 2008 A family of GHF5 endo-1,4-beta-glucanases in the migratory plant-parasitic nematode *Radopholus similis*. Plant Pathology 57:581–90.

[ref007] Haegeman A., Vanholme B. and Gheysen G. 2009 Characterization of a putative endoxylanase in the migratory plant-parasitic nematode *Radopholus similis*. Molecular Plant Pathology 10:389–401. 19400841 10.1111/j.1364-3703.2009.00539.x PMC6640231

[ref008] Hoff K. J., Lange S., Lomsadze A., Borodovsky M. and Stanke M. 2016 BRAKER1: Unsupervised RNA-Seq-based genome annotation with GeneMark-ET and AUGUSTUS. Bioinformatics 32:767–9. 26559507 10.1093/bioinformatics/btv661 PMC6078167

[ref009] Huang X., Xu C.-L., Yang S.-H., Li J.-Y., Wang H.-L., Zhang Z.-X., Chen C. and Xie H. 2019 Life-stage specific transcriptomes of a migratory endoparasitic plant nematode, *Radopholus similis* elucidate a different parasitic and life strategy of plant parasitic nematodes. Scientific Reports 9:6277. 31000750 10.1038/s41598-019-42724-7 PMC6472380

[ref010] Jacob J. E., Vanholme B., Van Leeuwen T. and Gheysen G. 2009 A unique genetic code change in the mitochondrial genome of the parasitic nematode *Radopholus similis*. BMC Research Notes 2:192. 19778425 10.1186/1756-0500-2-192 PMC2761399

[ref011] Jacob J., Mitreva M., Vanholme B. and Gheysen G. 2008 Exploring the transcriptome of the burrowing nematode *Radopholus similis*. Molecular Genetics and Genomics 280:1–17. 18386064 10.1007/s00438-008-0340-7

[ref012] Kumar S., Jones M., Koutsovoulos G., Clarke M. and Blaxter M. 2013 Blobology: exploring raw genome data for contaminants, symbionts and parasites using taxon-annotated GC-coverage plots. Frontiers in Genetics 4:237. 24348509 10.3389/fgene.2013.00237 PMC3843372

[ref013] Kurtz S., Phillippy A., Delcher A. L., Smoot M., Shumway M., Antonescu C. and Salzberg S. L. 2004 Versatile and open software for comparing large genomes. Genome Biology 5:R12. 14759262 10.1186/gb-2004-5-2-r12 PMC395750

[ref016] Nurk S., Meleshko D., Korobeynikov A. and Pevzner P. A. 2017 metaSPAdes: a new versatile metagenomic assembler. Genome Research 27:824–34. 28298430 10.1101/gr.213959.116 PMC5411777

[ref017] Simão F. A., Waterhouse R. M., Ioannidis P., Kriventseva E. V. and Zdobnov E. M. 2015 BUSCO: assessing genome assembly and annotation completeness with single-copy orthologs. Bioinformatics 31:3210–2. 26059717 10.1093/bioinformatics/btv351

